# Effect of the trap crop, *Solanum sisymbriifolium*, on *Globodera pallida*, *Globodera tabacum*, and *Globodera ellingtonae*

**DOI:** 10.21307/jofnem-2019-030

**Published:** 2019-05-27

**Authors:** L. M. Dandurand, I. A. Zasada, J. A. LaMondia

**Affiliations:** 1 Plant, Soil, and Entomological Sciences Department, 875 Perimeter Drive MS 2329, University of Idaho, Moscow, Idaho, 83844; 2 USDA-ARS Horticultural Crops Research Laboratory, 3420 NW Orchard Avenue, Corvallis, OR, 97330; 3 Connecticut Agricultural Experiment Station Valley Laboratory, 153 Cook Hill Road, Windsor, CT, 06095

**Keywords:** *Globodera* spp, Trap crop, Potato, Potato cyst nematode, *Solanum sisymbriifolium.*

## Abstract

The effect of the nematode trap crop *Solanum sisymbriifolium* was assessed against three *Globodera* spp., the potato cyst nematode *Globodera pallida* (in Idaho), the recently described *Globodera ellingtonae* (in Oregon), and the tobacco cyst nematode *Globodera tabacum* (in Connecticut) in field trials. At all locations the ability of *S. sisymbriifolium* to reduce *Globodera* encysted second-stage juveniles (J2) in egg densities compared to fallow was considered. For *G. ellingtonae*, the impact of planting and termination dates of *S. sisymbriifolium* on final egg densities was also evaluated; and for *G. pallida*, the ability of the nematode to reproduce on potato (*Solanum tuberosum*) after exposure to *S. sisymbriifolium* was determined. Encysted J2 in egg densities of all three *Globodera* spp. declined from 25 to 68% after trap cropping with *S. sisymbriifolium*. For *G. pallida*, *S. sisymbriifolium* reduced final encysted J2 in egg density by 23 to 50% compared to the fallow treatment, and significantly decreased *G. pallida* reproduction on potato after exposure to *S. sisymbriifolium* by 99 to 100% compared to the fallow treatment (*P* < 0.0001). For *G. ellingtonae*, the planting date of *S. sisymbriifolium* in May or June did not impact final egg densities (*P* = 0.32). Rather, percentage reduction in *G. ellingtonae* encysted J2 in egg density was most influenced by the length of time to which nematodes were exposed to *S. sisymbriifolium*, with 30 and 81% reduction after 6 vs 12 wk of exposure, respectively (*P* < 0.0001). Similar levels of nematode reduction after *S. sisymbriifolium* were observed for *G. tabacum* after 12 to 14 wk of exposure to the trap crop; *G. tabacum* density changes consisted of a 114% increase after susceptible tobacco, a 65% decrease after resistant tobacco, and an 88% decrease after *S. sisymbriifolium* compared to bare soil. In conclusion, this research demonstrates the widespread applicability of *S. sisymbriifolium* in reducing a diversity of *Globodera* spp. present in the USA.

Species in the genus *Globodera* are production-limiting pests in a number of crops, and novel methods are required to combat these plant-parasitic nematodes. *Globodera pallida* (Stone, 1973) Behrens, 1975, a potato cyst nematode, is of worldwide regulatory concern, and one of the most economically important pests of potato causing in excess of 80% yield loss in infested fields (Talavera et al., 1998). First detected in the United States in 2006 (Hafez et al., 2007), the introduction and potential spread of *G. pallida* has serious implications for US potato production and export. Consequently, it is under domestic and foreign quarantine restriction and parallel state regulations. In addition to the unexpected detection of *G. pallida* in Idaho, a newly described *Globodera* species, *Globodera ellingtonae* (Handoo et al., 2012), was discovered in Oregon and Idaho (Skantar et al., 2011; Handoo et al., 2012). Potato is a host for *G. ellingtonae* (Zasada et al., 2013), but this nematode is not regulated because pathogenicity has not been established on potato (Zasada and Ingham, personal comm.). Similar to potato cyst nematodes, *G. tabacum*, (Lownsberry and Lownsberry, 1954), has long been an economically important pathogen of cigar wrapper tobacco in the eastern United States, and control options are difficult, expensive, and of limited availability (LaMondia, 1995a, b).

Cyst nematodes are among the most challenging plant pests to control. The relatively narrow host range of *Globodera* spp. suggests that crop rotation could be effective for their control. However, crop rotation is an ineffective control measure for *Globodera* spp. because of their nearly absolute requirement for a hatching factor (Clarke and Perry, 1977; Arntzen et al., 1993; Byrne et al., 1998). In the absence of a suitable host, spontaneous hatch of *Globodera* eggs is only 10 to 30% per year (Evans, 1993; Whitehead, 1995; Turner et al., 2006; Trudgill et al., 2014), and encysted J2 in eggs may persist in soil for decades, where they remain relatively dormant and resistant to chemical and biological stresses (LaMondia and Brodie, 1986; LaMondia, 2008, 1995b).

Due to the difficulty in controlling *Globodera* spp., soil fumigants and nematicides have been the main control method. A critical part of the eradication effort of *G. pallida* in Idaho has been the use of the soil fumigant methyl bromide. However, because the use of methyl bromide has been discontinued, currently infested fields in Idaho are fumigated with 1,3-dichloropropene, which is not as effective and is difficult to obtain. Strategies to control cyst nematodes without reliance on fumigants are urgently needed (Zasada et al., 2010). Plant resistance, when available, has been an effective control mechanism. However, because resistance in potato to *G. pallida* is limited, and regulations prohibit the growth of potato in infested fields, use of resistant potato as a trap crop for *G. pallida* in Idaho is of limited value. One alternative strategy may be the use of trap crops. Trap crops, such as potato or tobacco, which promote hatch of cyst nematodes, without allowing reproduction, can deplete their population densities (LaMondia and Brodie, 1986; Turner et al., 2006; LaMondia, 2008; Trudgill et al., 2014).

A non-potato trap crop for *G. pallida* and *G. rostochiensis* that has received attention is *Solanum sisymbriifolium* (Lam.) (Timmermans et al., 2007; Dias et al., 2012; Dandurand and Knudsen, 2016). *Solanum sisymbriifolium* is native to South America. Scholte (2000a, 2000b) first noted the potential of *S. sisymbriifolium* as a trap crop for control of potato cyst nematodes. Up to 75% hatch of *G. pallida*, one season after planting *S. sisymbriifolium* has been reported (Timmermans *et al.*, 2006), whereas a 2-yr study found hatch to vary between 60 and 80% (Scholte and Vos, 2000). This plant was shown to be nearly as effective as potato at inducing egg hatching, but was resistant to subsequent development and reproduction of both *G. pallida* and *G. rostochiensis* (Kooliyottil et al., 2016). Under greenhouse conditions comparing pre-planting of *S. sisymbriifolium* to *Hordeum vulgare* (barley), a non-host for *G. pallida*, *S. sisymbriifolium* effectively reduced *G. pallida* populations by 99% in a subsequent potato crop, even when initial nematode population densities were high (Dandurand and Knudsen, 2016).

The goal of this research was to evaluate the potential to implement a *S. sisymbriifolium* trap crop for controlling three *Globodera* spp., *G. pallida*, *G. tabacum*, and *G. ellingtonae* in three US locations. Research was undertaken in diverse environments in which these nematodes are found and are economically important. Specific objectives of this research were to (i) evaluate the efficacy of *S. sisymbriifolium* in reducing egg population densities of *G. pallida*, *G. tabacum*, and *G. ellingtonae*, (ii) determine how planting date and planting duration of *S. sisymbriifolium* affects its trap cropping abilities on *G. ellingtonae* egg densities, and (iii) determine the reproductive potential of *G. pallida* on potato after cropping with *S. sisymbriifolium*.

## Materials and methods

Experiments were conducted in three locations in the United States where the specific *Globodera* spp. is already present. Because of the quarantine status of *G. pallida* it is only allowed to rear, maintain, and conduct experiments on this nematode in an USDA-APHIS approved facility. All experiments on *G. pallida* were conducted at the University of Idaho, Moscow, ID. Experiments on *G. ellingtonae* and *G. tabacum* were conducted at USDA-ARS, Corvallis, OR, or at the Connecticut Agricultural Experiment Station, Windsor, CT, respectively.

### Rearing of *Globodera* used in field experiments

The *G. pallida* population was originally isolated from an infested ﬁeld in Shelley, ID. The population was reared under greenhouse conditions on the susceptible potato ‘Désirée’ in clay pots (15-cm diam.) ﬁlled with a sterilized sandy loam soil and sand (2:1) mix with night temperature of 10°C and day temperature of 18°C, at a 16:8-h (day:night) photoperiod for all experiments. Species conﬁrmation was achieved through morphological and molecular identiﬁcation (Skantar Skantar et al., 2007). After culturing the nematodes for 16 wk, cysts for experimental use were extracted from soil using Fenwick’s (1940) can method (Fenwick, 1940) and picked by hand under a stereomicroscope (Leica Microsystems, Wetzlar, Germany). To estimate the number of encysted eggs, 10 cysts were crushed with a rubber stopper, eggs were washed into a container, adjusted to a desired volume, and eggs/ml were determined under an inverted microscope (Leica Microsystems). Cysts were placed in sealed pouches (2.54 cm^2^) made of wear resistant nylon mesh (248.92 µm opening) from McMaster-Carr (Elmhusrt, IL), which allows migration of the hatched J2 through the nylon mesh, but retains the cyst for ease of encysted egg counts at the end of the growing season. In 2015, cysts contained approximately 375 eggs/cyst and each cyst bag contained 25 cysts; in 2016, cysts contained approximately 410 eggs/cyst and each cyst bag contained 23 cysts.

*Globodera ellingtonae* inoculum was produced at Oregon State University Central Oregon Agricultural Research Center (OSU COARC), Powell Butte, OR using the resident *G. ellingtonae* population. In the year prior to the experiment, susceptible potato “Russet Burbank” was inoculated with *G. ellingtonae* encysted eggs in 22-liter pots (Grip Lip 2800; Nursery Supplies Inc., McMinnville, OR) buried in the field. The plants were allowed to grow the entire season, naturally senesce, and the pots containing nematodes remained in the field over the winter. The following spring, pots were removed from the ground and soil was dried and sieved to remove large debris. To create soil with high cyst densities, the floating fraction from dried soil was extracted, allowed to dry for 2 to 3 d, and then mixed back into soil containing cysts. The egg density of this soil was then determined by extracting cysts from 50 g of soil from five random samples using an USDA cyst extractor (Spears, 1968). Then, cysts were hand-picked and counted. Cysts were crushed as described above and eggs were counted. Mesh bags of soil containing cysts were constructed by placing 35 g of the cyst-infested-soil in a 10-cm square of 250-µm nylon mesh heat sealed to close (Jores Technology, Sunrise, FL). In 2014, sealed bags contained ~50 cysts with a mean of 307 ± 34 eggs per cyst, and in 2015 sealed bags contained ~75 cysts with a mean of 311 ± 26 eggs per cyst.

*Globodera tabacum* inoculum was derived from soil naturally infested with various densities of *G. tabacum* after a tobacco crop. Initial densities were determined by extracting cysts from 500 cm^3^ soil using a modified Fenwick can. Extracted cysts were crushed and viable eggs and J2 counted as described above. Densities ranged from 4 to 445 (mean = 125) viable encysted J2 per cm^3^ in 2015 and from 1 to 328 (mean = 59) viable encysted J2 per cm^3^ soil in 2016.

### Effect of *S. sisymbriifolium* on *G. pallida* egg density and reproduction

*Globodera pallida* populations in infested fields in Idaho are low in density, highly aggregated, and variable in both viability and in egg numbers (T. Gresham, personal communication). Increasing field populations by growing potato is not possible because of restrictions in place by USDA-APHIS. To demonstrate the potential of S*. sisymbriifolium* under field conditions, greenhouse-reared *G. pallida* cysts were used (as described above) and a containment protocol was developed and approved by USDA-APHIS and ISDA to prevent the possible escape of introduced cysts. To achieve containment, cysts were placed in sealed bags as described above.

Microplot trials were conducted in Shelly, ID in 2015 and 2016. Microplots were prepared by drilling four 1-cm holes in the bottom of a bucket (18.9 liter) and sealing nylon mesh (same as used for cyst bags) over each hole with silicon caulking. To prevent any possible escape of *G. pallida* into the surrounding field soil, each bucket was then placed inside of another bucket of the same size that had not been drilled, and the unit was placed into holes in the soil created by a backhoe. Each bucket was filled with 15 kg of a Bannock silt loam field soil. Cysts (2.5 encysted eggs/g soil) contained in nylon mesh bags were placed 15-cm below the soil surface and covered with soil. Microplots contained five cyst bags each, with one bag retrieved for estimation of encysted eggs and four bags remained in the microplots for the potato bioassay (see below). The nylon mesh bags were attached to stakes for ease of retrieval. *Solanum sisymbriifolium* seeds (Accession PI 381291) were obtained from Chuck Brown, USDA-ARS, Prosser, WA, and 4-wk-old transplants (approximately 8-cm in height, five plants per microplot) were planted; a bare soil treatment was used as the control. Treatments (with or without *S. sisymbriifolium*) were replicated six times and arranged in a randomized block design in both experiments. At the termination of the experiment, *S. sisymbriifolium* tops were cut at the crown of the plant, but roots remained in place.

To assess treatment effects after 10 and 12 wk in 2015 and 2016, respectively, remaining encysted egg numbers were determined, and a potato bioassay was conducted. The number of encysted eggs was determined by releasing eggs from 10 cysts for each replicate into 0.5 ml water, and enumerating all eggs in a 100 µl aliquot using a stereomicroscope (Leica Microsystems). The microplot buckets containing the field soil and the *G. pallida* population from the remaining four cyst bags were placed at 4°C for an 8-wk chilling period (Perry and Gaur, 1996; Perry and Moens, 2011; Palomares-Ruis et al., 2013). To determine treatment effects on multiplication of *G. pallida*, a potato bioassay was conducted under greenhouse conditions for an additional 16 wk. For all bioassay experiments, potato ‘Désirée’ grown from tissue culture plantlets in standard media (Murashige and Skoog, 1962) and approximately 8-cm in height plantlets were transplanted directly into the 18.9-liter bucket transported from the field which contained field soil infested with cysts as described above. All plants were maintained at even moisture by watering daily and fertilizing with Jacks Classic 20-20-20 (N-P-K) all-purpose fertilizer (J. R. Peters Inc., Allentown, PA) three-times-per-week (15 g/liter). After 16 wk, *G. pallida* cysts were extracted from soil and eggs enumerated as described above. For treatments where new cysts were produced, the reproductive rate was determined as final egg population (*Pf)*/initial egg population (*Pi*).

Data were analyzed by analysis of variance (ANOVA) using the general linear model statement in Statistical Analysis Software (SAS) (SAS Institute Inc., Cary, NC). To meet assumptions for an analysis of variance (ANOVA), square root or arcsine transformations were used to ensure a normal distribution and constant variation of the count and hatching data, respectively. Means were separated at *P* ≤ 0.05.

### Influence of planting and termination date of *S. sisymbriifolium* on *G. ellingtonae* egg densities

All field trials were carried out at the OSU COARC, Powell Butte, OR. The soil at this location is a Redmond ashy sandy loam. In 2014, an experiment was conducted to determine the impact of *S. sisymbriifolium* planting and termination date on *G. ellingtonae* egg densities. The experiment was a 2 × 4 × 3 factorial experiment arranged in a randomized block design with six replications per treatment combination. The factors consisted of two plant types (*S. sisymbriifolium* or fallow) × four planting dates (May 19, 2014 and at three-3-wk intervals thereafter) × three termination dates (3, 6, and 9 wk after planting). The entire area of the trial was 7.4 × 11.8 m^2^ and each plot was 40 × 60 cm^2^ with 1 m between plots. Prior to establishing the experiment, the area received 1,344 kg/ha 16-16-16 (N-P-K) prior to shallow cultivation. At each planting date, three bags containing cysts (as described above) were buried 45 cm deep in each plot and then 12 4-wk-old *S. sisymbriifolium* transplants were planted in each plot spaced 10-cm from bags containing cysts. Fallow plots only received bags containing cysts. The trial area was overhead sprinkler irrigated, received no additional fertilizer, and was hand weeded as necessary. At each termination date, one cyst bag was removed from six replicates of *S. sisymbriifolium* (with plants being left in the field) or fallow treatments and the bags were transported to the laboratory the same day as collection and stored at 4°C until being processed within 2 d. To extract cysts, the bags were cut open and all contents placed in a 2-liter beaker, vigorously mixed with ~1 liter of water, and cysts were collected by pouring the solution over 250-µm sieves. Collected cysts were washed onto filter paper, dried, and stored at room temperature until further processing. Cysts from each sample were collected and eggs per cyst enumerated as described above.

In 2015, another experiment was conducted at Powell Butte, OR to examine the impact of *S. sisymbriifolium* planting and termination date on *G. ellingtonae* egg densities. The experiment was a 2 × 2 × 3 factorial with treatments arranged in a completely randomized design with five replicates per treatment combination. The factors consisted of two plant types (*S. sisymbriifolium* and fallow), two planting dates (June 8 and 29, 2015), and three termination dates (6, 9, and 12 wk after planting). The entire area of the trial was 2.5 × 12.5 m^2^ and each plot was 20 × 20 cm^2^ with 1 m between plots. Prior to establishing the experiment, the area was prepared as described for the 2014 experiment. In *S. sisymbriifolium* plots, four 4-wk-old *S. sisymbriifolium* transplants were planted around bags containing cysts at a distance of 10 cm from the bag. The trial area was overhead sprinkler irrigated, received no additional fertilizer, and was hand weeded as necessary. At each termination date, five plots of each *S. sisymbriifolium* and fallow were sampled. In *S. sisymbriifolium* plots, the plants were dug from the ground. The cyst bags were collected and processed as described above.

Data from each trial conducted in 2014 and 2015 were analyzed separately. Data were analyzed for effects of treatment (*S. sisymbriifolium* or fallow), planting date, and termination date and interactions using PROC MIX ANOVA with trap crop treatment, planting date, and termination data as fixed effects and replication block as a random effect. Treatments means were separated using Dunnett’s adjustment for multiplicity (*P* ≤ 0.05). All analyses were performed using JMP 9.1 (SAS Institute Inc.).

### Effect of *S. sisymbriifolium* on *G. tabacum* egg density

Field research was conducted in 2015 and 2016 at the Connecticut Agricultural Experiment Station, Valley Laboratory Research Farm, Windsor, CT in an established shade tent. In total, 65 field microplots (1-m diam.) containing Merrimac fine sandy loam soil (71.8% sand, 23.0% silt, 5.2% clay, pH 6.0, 4.0% organic matter) naturally infested with various densities of *G. tabacum* were used. Treatments were blocked across low to high *G. tabacum* densities. Microplots were transplanted on June 26, 2015 and July 8, 2016 with nematode-susceptible shade tobacco (*N. tabacum* ‘8212’ in 2015, and ‘O-40’ in 2016), nematode-resistant broadleaf tobacco ‘B2’ (LaMondia, 2012), or *S. sisymbriifolium*. In 2016, treatments were expanded to include eastern black nightshade (*Solanum ptychanthum*) and a cultivated bare fallow. In both years, microplots were fertilized with cottonseed meal based 8-3-8 (N-P-K) tobacco fertilizer (78.5 kg N/ha) prior to planting and side-dressed twice at 2 and 4 wk after transplanting with additional tobacco fertilizer at the same rate to result in a total of 235 kg N/ha. *Globodera tabacum* densities were determined before planting and again after harvest by collecting 10 soil cores of 1.5-cm diam. taken to 15-cm in depth from each microplot. Plants were stalk cut, removed and soil within each microplot were cultivated with a small shovel that was cleaned between microplots to mix roots and soil on September 21, 2015 and October 7, 2016 prior to taking soil cores on September 22, 2015 and October 18, 2016. Soil was dried and 250 cm^3^ soil per microplot extracted using a modified Fenwick can (Spears, 1968). As soil in microplots had been infested with *G. tabacum* for decades, large numbers of cysts were always present. Cysts were dried and then separated from root debris by flotation in acetone in a milk filter sock. Cysts were rehydrated for 24 hr in water in a test tube, crushed using a tissue grinder and the number of viable encysted J2 per cm^3^ soil determined by counting two 1-ml aliquots per microplot. Changes in population density were expressed as the *Pf/Pi* ratio. As data were non-normal, initial and final nematode densities per cm^3^ soil were log transformed to normalize data and the differences between *Pf* and *Pi* logs were analyzed by ANOVA and means separated by Fisher’s LSD multiple comparison tests (*P* ≤ 0.05).

## Results

### Effect of *S. sisymbriifolium* on *G. pallida* egg density and reproduction

Exposing *G. pallida* to *S. sisymbriifolium* for 10 or 12 wk in 2015 and 2016, respectively, reduced encysted egg densities compared to fallow (Tables 1 and 2). In 2015, the number of *G. pallida* eggs remaining after a 10-wk exposure to *S. sisymbriifolium* was approximately 23% less than from the fallow treatment (Table 1). However, the percentage viability of that population as determined by microscopic observation did not differ significantly whether eggs were exposed to *S. sisymbriifolium* or bare fallow soil for 10 wk. In 2016, a 12-wk exposure to *S. sisymbriifolium* resulted in a 50% reduction of eggs/cyst compared to the fallow treatment (Table 2). As observed in 2015, *S. sisymbriifolium* had no impact on the viability of the remaining population of J2 compared to the fallow treatment.

**Table 1.** Effect of *Solanum sisymbriifolium* on *Globodera pallida* egg density and viability and on subsequent reproduction on potato in 2015.

	Remaining eggs/cysts	Egg viability (%)	Progeny cyst (no./kg soil)	Encysted eggs (eggs/g soil)	*Pf* ^a^ (eggs/g soil)	*Pf/Pi* ^b^ (eggs/g soil)
Treatment	After 10 wk^c^		Reproduction on potato^d^			
Fallow	299^e^	57.0	36	196	6.9	2.8
*S. sisymbriifolium*	230	58.1	4	10	0.02	0.01
*P*-value^f^	0.10	0.92	0.0001	0.0001	0.0001	0.0001

Note: ^a^ *Pf*=final egg population density; ^b^ *Pf/Pi*=final egg population density/initial egg population density (*Pi *); ^c^eggs remaining in cysts initially used for soil infestation were measured 10 wk after infesting into bare soil or S. sisymbriifolium. Soil was infested with a *Pi* of 2.5 encysted eggs/g soil; ^d^reproduction was assessed after a second 16-wk cycle where all treatments were planted to potato; ^e^values are the mean of six replicates; ^f^ *P*-value was determined by analysis of variance.

**Table 2.** Effect of *Solanum sisymbriifolium* on *Globodera pallida* egg density and viability and on subsequent reproduction on potato in 2016.

	Remaining eggs/cysts	Egg viability (%)	Progeny cyst (no./kg soil)	Encysted eggs (eggs/g soil)	*Pf* ^a^ (eggs/g soil)	*Pf/Pi* ^b^ (eggs/g soil)
Treatment	After 12 wk^c^		Reproduction on potato^d^			
Fallow	162^e^	71.4	20	187	3.5	1.4
*S. sisymbriifolium*	81	66.4	0	0	0	0
*P*-value^f^	0.002	0.19	0.0006	0.0001	0.0001	0.0001

Note: ^a^ *Pf* = final egg population density; ^b^ *Pf/Pi* = final egg population density/initial egg population density (*Pi*); ^c^eggs remaining in cysts initially used for soil infestation were measured 12 wk after infesting into bare soil or S. sisymbriifolium. Soil was infested with a *Pi* of 2.5 encysted eggs/g soil; ^d^reproduction was assessed after a second 16-wk cycle where all treatments were planted to potato; ^e^values are the mean of six replicates; ^f^ *P*-value was determined by analysis of variance.

A potato bioassay was conducted to evaluate the impact of *S. sisymbriifolium* on reproduction of *G. pallida* on a subsequent potato crop. At the end of the 16 wk bioassays, the number of progeny cysts, eggs/cyst, *Pf* (eggs), and *Pf*/*Pi* was significantly lower in the potato-following-*S. sisymbriifolium* treatment compared to potato-following-fallow treatment in both experiments (Tables 1 and 2). In 2015, *S. sisymbriifolium* had a significant impact on reproduction of *G. pallida* with 88% fewer progeny cysts from potato-following-*S. sisymbriifolium* compared to potato-following-fallow treatment (Table 1). Not only were there fewer cysts, but newly produced cysts also contained 95% fewer eggs when produced on potato after *S. sisymbriifolium* compared to cysts produced on potato following the fallow treatment (Table 1). The impact of exposure of *S. sisymbriifolium* to *G. pallida* reproduction on a potato crop following *S. sisymbriifolium* in 2016 was similar to that observed in 2015. In 2016, reproduction of *G. pallida* was completely eliminated on potato following *S. sisymbriifolium*, although *G. pallida* reproduction occurred on potato following the fallow treatment (Table 2).

### Influence of planting and termination date of *S. sisymbriifolium* on *G. ellingtonae* egg densities

In both years, planting date as a main effect or the interaction of planting date with treatment and termination date were not significant (*P* ≤ 0.1). Therefore, only the significant main effects of treatment and termination date, as well as this interaction, were considered. In 2014, the main effects of treatment (fallow and *S. sisymbriifolium*) and termination date (3, 6, and 9 wk after planting), as well as the interaction between treatment and termination date, were significant (*P* ≤ 0.001) (Table 3). When the interaction was considered, there were 50 and 65% fewer eggs recovered from *S. sisymbriifolium* at 6 and 9 wk after planting, respectively, compared to the early collection at 3 wk after planting (Table 3). A similar reduction over time in egg densities under fallow was not observed.

**Table 3.** Effect of treatment (bare ground or *Solanum sisymbriifolium*) and termination date after treatment establishment on *Globodera ellingtonae* egg densities in soil at Powell Butte, Oregon in 2014 and 2015.

	Termination date (weeks after S. sisymbriifolium planting)^a^			
Treatment	3	6	9	12
*G. ellingtonae* eggs/cyst^b^				
2014				
*S. sisymbriifolium*	196 a^c^	98 b	69 b	nt^d^
Fallow	209 a	195 a	173 a	nt
2015				
*S. sisymbriifolium*	nt	218 b	108 c	58 d
Fallow	nt	271 a	261 a	179 b

Note: ^a^Treatments were established on May 19, 2014 and June 8, 2015; ^b^initial density in 2014 was 30 ± 734 eggs/cyst with approximately 50 cysts, and in 2015 was 31126 with approximately 75 cysts; ^c^values are the means of six observations. All values in the same year followed by the same letter are not significantly different according to Dunnett’s adjustment for multiplicity (P ≤ 0.05); ^d^nt = not tested.

In 2015, similar to 2014, the main effect of treatment (fallow and *S. sisymbriifolium*) and termination date (6, 9, and 12 wk after planting), as well as the interaction between treatment and termination date, were significant (*P* ≤ 0.001). As in 2014, the longer *G. ellingtonae* eggs were exposed to *S. sisymbriifolium*, the greater the reduction in egg number; this is shown by the interaction of treatment and termination date (Table 3). By 12 wk after planting, egg densities under *S. sisymbriifolium* and fallow were significantly lower than that observed at 9 wk after planting. This resulted in an 82% reduction in initial *G. ellingtonae* egg densities and 68% fewer eggs than in the corresponding fallow treatment. At the other termination dates, 6 and 9 wk after planting, there were always fewer eggs recovered from *S. sisymbriifolium* compared to fallow on the same termination date (Table 3).

### Effect of *S. sisymbriifolium* on *G. tabacum* egg density

*Globodera tabacum* reproduction in field microplots, as determined by *Pf*/*Pi*, varied among tobacco varieties and the trap crop treatments in 2015 (Table 4). There was a substantial increase in *Pf/Pi* on susceptible tobacco compared to planting resistant tobacco (Table 4). Population densities of *Globodera tabacum* were reduced by 62 and 86% under resistant tobacco and *S. sisymbriifolium*, respectively, after 12.5 wk compared to densities under resistant tobacco. In 2016, resistant tobacco, *S. sisymbriifolium*, and fallow reduced population densities of *G. tabacum* by 64, 88, and 50%, respectively. Among which, *S. sisymbriifolium* resulted in the lowest *Pf/Pi* of *G. tabacum* (Table 4).

**Table 4.** Influence of nematode-susceptible or resistant tobacco, *Solanum ptychanthum*, *S. sisymbriifolium*, fallow on *Globodera tabacum* population change over one season in field microplots.

	2015			2016		
Treatment	*Pi* ^a^	*Pf* ^a^	*Pf/Pi* ^a^	*Pi*	*Pf*	*Pf/Pi*
Susceptible tobacco	90.6^b^	193.6	2.9 a^c^	64.0	173.8	2.8 a
Resistant tobacco	103.8	36.4	0.4 b	82.6	29.7	0.3 b
*S. ptychanthum*	nt^d^	nt	nt	77.3	335.1	6.6 a
*S. sisymbriifolium*	91.1	10.6	0.1 c	75.6	9.3	0.2 c
Fallow	nt	nt	nt	86.4	43.4	0.56 b
*P*-value	0.89	0.0001	0.0001	0.86	0.0001	0.0001

Note: ^a^Pf/Pi = final egg population density (Pf)/initial egg population density (Pi). Data were log transformed and differences between final and initial populations analyzed by analysis of variance. Means were separated by Fishers LSD multiple comparison test; ^b^values are the means of 20 replicate plots in 2015 and 12 or 13 replicates in 2016. Values followed by the same letter are not significantly different according to Fisher’s LSD multiple comparison tests (P ≤ 0.05); ^c^numbers within columns followed by the same letter are not significantly different; ^d^nt = not tested.

As an overall summary of this project, encysted egg populations of *G. pallida*, *G. ellingtonae*, and *G. tabacum* declined from 25 to 68% after cropping with *S. sisymbriifolium* compared to the fallow treatment. All three species were impacted by the trap crop and the magnitude of the decline increased with increasing time of exposure to the trap crop.

## Discussion

This is the first report on the efficacy of *S. sisymbriifolium* for the management of *Globodera* spp. in the United States. *Solanum sisymbriifolium* has received attention in Europe for the management of *G. pallida* and *G. rostochiensis* (Scholte and Vos, 2000; Scholte, 2000a, 2000b; Timmermans et al., 2006, 2009). Current research demonstrates that *S. sisymbriifolium* was an effective trap crop for a diversity of *Globodera* spp. found in the US; the potato cyst nematode *G. pallida*, the recently described *G. ellingtonae*, and the related tobacco cyst nematode *G. tabacum*. To our knowledge, this is the first report on the ability of *S. sisymbriifolium* to reduce encysted J2 in egg densities of *G. ellingtonae* and *G. tabacum*. Levels of reduction for G. *ellingtonae* were consistent over the years, but at the end of the growing season were slightly higher than that of *G. pallida* and *G. tabacum*. It has been previously demonstrated that *G. ellingtonae* is more similar to *G. rostochiensis* than *G. pallida* when hatching dynamics and temperature requirements for development were considered (Zasada et al., 2013; Phillips et al., 2017). The effects of *S. sisymbriifolium* on *G. pallida* or *G. rostochiensis* have been reported to be in a similar range to what we observed, with 30 to 47% reduction in egg densities during a 6- to 13-wk period and up to 80% reduction after 14 wk in another study (Scholte and Vos, 2000; Scholte, 2000b). The population of *G. tabacum* in field microplots was decreased by 80 to 90% after *S. sisymbriifolium*, significantly more than after planting resistant tobacco, currently used to manage this nematode.

Similar to previous reports, the length of time to which a *S. sisymbriifolium* trap crop is grown will influence efficacy in reducing densities of *Globodera* spp. eggs. Timmermans et al. (2006) found that reduction in *G. pallida* egg number increased with length of exposure with a 47% reduction in eggs 6 wk after planting and a 75% reduction in eggs 21 wk after planting. In another study, potato stimulated hatch of G*. rostochiensis* more than *S. sisymbriifolium*, but this difference decreased with longer growth period (Scholte and Vos, 2000). Results from this study supported that *S. sisymbriifolium* should be allowed to grow for as long as the cropping practice allowed to maximize its trap cropping effect against *Globodera* spp. For *G. ellingtonae*, termination time after planting *S. sisymbriifolium* had a greater influence on egg densities than the planting date. In general, for both *G. ellingtonae* and *G. pallida*, the reduction in number of eggs/cyst increased with time with the greatest reduction in encysted eggs occurring when duration of the trap crop growth was longer. However, there may be a point after planting when no additional reduction will be achieved, as was observed for *G. tabacum* with similar percent reductions in initial egg densities, 90%, at 12 and 14 wk.

The ability of *S. sisymbriifolium* as a trap crop to reduce population densities of *G. pallida* prior to potato planting is very encouraging in Idaho for a potato industry that has zero tolerance for *G. pallida*. Conventional crop rotation with partially resistant potato varieties against *G. pallida* is only effective when combined with a nematicide (Turner et al., 2006). Trudgill et al. (2014) reported that a minimum of 8 yr of fallow is required to manage *G. pallida*, and the partially resistant potato variety ‘Santé’ still resulted in a 5-or 30-fold increase in *G. pallida* multiplication. The very low rate of *G. pallida* reproduction on susceptible potato after a rotation with *S. sisymbriifolium* makes this trap crop potentially useful for control of this nematode, despite the loss of income from planting a trap crop for one season. In Idaho, regulatory action by USDA-APHIS prohibits the planting of any potato crop in an infested field (USDA-APHIS, 2009).

Although evidence supports the observation that *S. sisymbriifolium* reduces potato cyst nematode populations through stimulus of hatching while not supporting nematode development (Scholte, 2000b; Timmermans et al., 2006), other factors may also be involved (Dandurand and Knudsen, 2016; Dias et al., 2012). One possibility may be that hatching stimulus from roots of *S. sisymbriifolium* continues even after removal of shoots so that hatch of *G. pallida* may have continued after termination of the first plant cycle with *S. sisymbriifolium*. Alternatively, *S. sisymbriifolium* may contain glyco-alkaloid that could potentially be nematicidal (Dias et al., 2012) which may lead to a decrease in densities of *G. pallida*. Further research is necessary to investigate this phenomenon.

The efficacy of *S. sisymbriifolium* in reducing egg densities of three *Globodera* spp. spanned across three geographically and climatologically diverse environments. Timmermans et al. (2009) modeled the potential of *S. sisymbriifolium* as a trap crop for *G. rostochiensis* and *G. pallida* across western and central Europe. In their analysis they considered three different scenarios: (i) a zone with insufficient growth, independent of planting date, (ii) a zone with potentially sufficient growth in the crop if the crop is allowed to grow for the whole season, and (iii) a zone with sufficient growth early or late in the growing season, allowing for a double-cropping situation. All of the sites in the USA where this research was conducted are between the 41° and 44° parallels and would fall into scenario (ii). These would be similar to the parameters of growth and planting date that were purposed for southern France and northern Italy (Timmermans et al., 2009). In North America, locations further north such as Quebec, where *G. rostochiensis* has been found, were determined to be unsuitable for growing a *S. sisymbriifolium* trap crop (Bélair et al., 2016).

In summary, use of a *S. sisymbriifolium* trap crop was effective in reducing egg densities of *G. pallida*, *G. ellingtonae*, and *G. tabacum* within a single growing season in the USA. In addition to serving as a trap crop for these nematodes, it appears that a secondary mechanism of suppression may also be active, with eggs exposed to root exudates of *S. sisymbriifolium* reduced in their ability to parasitize and complete development on a subsequent potato crop. From a practical application perspective, this research demonstrated that a *S. sisymbriifolium* trap crop should be kept in the field as long as possible after planting to maximize the reduction in *Globodera* egg density. Therefore, in Idaho, *S. sisymbriifolium* may play an important role in an integrated plan to eliminate *G. pallida* from infested fields.

## References

[ref001] Arntzen F. K., Visser J. H. M. and Hoogendorn J. 1993 Hatching of Globodera pallida juveniles by diffusate of potato genotypes, differing in tolerance to G. pallida. Annals of Applied Biology 123: 83–91.

[ref002] Bélair G., Dauphinais N. and Mimee B. 2016 Evaluation of cultural methods for the management of the golden nematode (Globodera rostochiensis) in Quebec, Canada. Canadian Journal of Plant Pathology 38: 209–217.

[ref004] Byrne J., Twomey U., Maher N., Devine K. J. and Jones P. W. 1998 Detection of hatching inhibitors and hatching factor stimulants for golden potato cyst nematode, Globodera rostochiensis, in potato root leachate. Annals of Applied Biology 132: 463–472.

[ref005] Clarke A. J. and Perry R. N. 1977 Hatching of cyst-nematodes. Nematologica 23: 350–368.

[ref006] Dandurand L. M. and Knudsen G. R. 2016 Effect of the trap crop Solanum sisymbriifolium and two biocontrol fungi on reproduction of the potato cyst nematode, Globodera pallida. Annals of Applied Biology 169: 180–189.

[ref007] Dias M. C., Conceição L., Abrantes I. and Cunha M. J. 2012 Solanum sisymbriifolium – a new approach for the management of plant-parasitic nematodes. European Journal of Plant Pathology 133: 171–179.

[ref008] Evans K. 1993 Reviews: new approaches for potato cyst nematode management. Nematropica 23: 221–231.

[ref009] Fenwick D. W. 1940 Methods for the recovery and counting of cysts of Heterodera schachtii from soil. Journal of Helminthology 18: 155–172.

[ref010] Hafez S. L., Sundararaj P., Handoo Z. A., Carta L. K. and Chitwood D. J. 2007 First report of the pale cyst nematode, Globodera pallida, in the United States. Plant Disease 91: 325. 10.1094/PDIS-91-3-0325B 30780577

[ref011] Handoo Z. A., Carta L. K., A. M. and Chitwood D. J. 2012 Description of Globodera ellingtonae n. sp. (Nematoda: Heteroderidae) from Oregon. Journal of Nematology 44: 40–57. 23483076 PMC3593265

[ref012] Kooliyottil R., Dandurand L. M., Govindan B. N. and Knudsen G. R. 2016 Microscopy method to compare cyst nematode infection of different plant species. Advances in Bioscience and Biotechnology 7: 311–318.

[ref013] LaMondia J. A. 1995a Shade tobacco yield loss and Globodera tabacum population changes in relation to initial nematode density. Journal of Nematology 27: 114–119. 19277269 PMC2619594

[ref014] LaMondia J. A. 1995b Hatch and reproduction of Globodera tabacum in response to tobacco, tomato, or black nightshade. Annals of Applied Nematology 27: 382–386. PMC2619612 19277303

[ref015] LaMondia J. A. 2008 Early crop root destruction for management of tobacco cyst nematodes. Journal of Nematology 40: 26–29. 19259515 PMC2586525

[ref016] LaMondia J. A. 2012 Registration of B2 Connecticut broadleaf cigar-wrapper tobacco resistant to Fusarium wilt, Tobacco Mosaic Virus, cyst nematodes and blue mold. Journal of Plant Registrations 7: 58–62.

[ref017] LaMondia J. A. and Brodie B. B. 1986 The effect of potato trap crops and fallow on decline of Globodera rostochiensis. Annals of Applied Biology 108: 347–352.

[ref003] Lownsberry B. F. and Lownsberry J. W. 1954 Heterodera tabacum new species, a parasite of solanaceous plants in Connecticut. Proceedings of the Helminthological Society of Washington 21: 42–47.

[ref018] Murashige T. and Skoog F. 1962 A revised medium for rapid growth and bio assays with tobacco tissue cultures. Physiologia Plantarum 15: 473–497.

[ref019] Palomares-Ruis J. E., Jones J. T., Cock P. J., Castillo P. and Blok V. C. 2013 Activation of hatching in diapaused and quiescent Globodera pallida. Parasitology 140: 445–454. 23253858 10.1017/S0031182012001874

[ref020] Perry R. N. and Gaur H. S. 1996 Host plant inﬂuences on the hatching of cyst nematodes. Fundamental & Applied Nematology 19: 505–510.

[ref021] Perry R. N. and Moens M. 2011 Survival of parasitic nematodes outside the host in Perry R. and Wharton D. (Eds), Molecular and physiological basis of nematode survival, CABI Publishing, Wallingford:1–27.

[ref022] Phillips W. P., Kitner M. and Zasada I. A. 2017 Developmental dynamics of Globodera ellingtonae in field-grown potato. Plant Disease 101: 1182–1187. 30682969 10.1094/PDIS-10-16-1439-RE

[ref023] Scholte K. 2000a Growth and development of plants with potential for use as trap crops for potato cyst nematodes and their effects on the numbers of juveniles in cysts. Annals of Applied Biology 137: 31–42.

[ref024] Scholte K. 2000b Screening of non-tuber bearing Solanaceae for resistance to and induction of juvenile hatch of potato cyst nematodes and their potential for trap cropping. Annals of Applied Biology 136: 239–246.

[ref025] Scholte K. and Vos J. 2000 Effects of potential trap crops and planting date on soil infestation with potato cyst nematodes and root-knot nematodes. Annals of Applied Biology 137: 153–164.

[ref026] Skantar A. M., Handoo Z. A., Carta L. K. and Chitwood D. J. 2007 Morphological and molecular identification of Globodera pallida associated with potato in Idaho Journal of Nematology 39: 133–144. 19259482 PMC2586493

[ref038] Skantar A. M., Handoo Z. A., Zasada I. A., Ingham R. E., Carta L. K. and Chitwood D. J. 2011 Morphological and Molecular Characterization of Globodera Populations from Oregon and Idaho Phytopathology 101: 480–491. 21391826 10.1094/PHYTO-01-10-0010

[ref027] Spears J. M. 1968 The golden nematode handbook, USDA Handbook 353, Washington, DC.

[ref039] Stone A. R. 1973 Heterodera pallida C.I.H. Descriptions of plant-parasitic nematodes 2: 17–18.

[ref028] Talavera M., Andreu M., Valor H. and Tobar A. 1998 Nematodos fitoparásiticos en áreas productoras de patata de Motril y Salobreña. Investigacion Agraria, Produccion y Proteccion Vegetales 13: 87–95.

[ref029] Timmermans B. G. H., Vos J. and Stomph T. J. 2009 The development, validation, and application of a crop growth model to assess the potential of Solanum sisymbriifolium as a trap crop for potato cysts nematodes in Europe. Field Crops Research 111: 22–31.

[ref030] Timmermans B. G. H., Vos J., Stomph T. J., Van Nieuwburg J. and Van der Putten P. E. L. 2006 Growth duration and root length density of Solanum sisymbriifolium (Lam.) as determinants of hatching of Globodera pallida (Stone). Annals of Applied Biology 148: 213–222.

[ref031] Timmermans B. G. H., Vos J., Van Nieuwburg J., Stomph T. J., Van der Putten P. E. L. and Molendijk P. G. 2007 Field performance of Solanum sisymbriifolium, a trap crop for potato cyst nematodes. I. Dry matter accumulation in relation to sowing time, location, season and plant density. Annals of Applied Biology 150: 89–97.

[ref032] Trudgill D. L., Phillips M. S. and Elliott M. J. 2014 Dynamics and management of the white potato cyst nematode Globodera pallida in commercial potato crops. Annals of Applied Biology 164: 18–34.

[ref033] Turner S. J., Martin T. J. G., McAleavey P. B. W. and Fleming C. C. 2006 The management of potato cyst nematodes using resistant Solanaceae potato clones as trap crops. Annals of Applied Biology 149: 271–280.

[ref034] USDA-APHIS 2009 Potato cyst nematode; quarantine and regulations AGENCY: animal and plant health inspection service, USDA. ACTION: Final rule.Federal Register 19374Vol. 74, No. 81, April 29.

[ref035] Whitehead A. G. 1995 Decline of potato cyst nematodes, Globodera rostochiensis and G. pallida, in spring barley microplots. Plant Pathology 44: 191–195.

[ref036] Zasada I. A., Peetz A. B., Wade N., Navarre R. A. and Ingham R. E. 2013 Host status of different potato (Solanum tuberosum) varieties and hatching in root diffusates of Globodera ellingtonae. Journal of Nematology 45: 195–201. 24115784 PMC3792837

[ref037] Zasada I. A., Halbrendt J. M., Kokalis-Burelle N., LaMondia J., McKenry M. V. and Noling J. W. 2010 Managing nematodes without methyl bromide. Annual Review of Phytopathology 48: 311–328. 10.1146/annurev-phyto-073009-114425 20455696

